# The zebrafish *goosepimples/myosin Vb* mutant exhibits cellular attributes of human microvillus inclusion disease^☆^^☆☆^

**DOI:** 10.1016/j.mod.2016.08.001

**Published:** 2016-11

**Authors:** Jaydeep Sidhaye, Clyde Savio Pinto, Shweta Dharap, Tressa Jacob, Shobha Bhargava, Mahendra Sonawane

**Affiliations:** a Department of Biological Sciences, Tata Institute of Fundamental Research, Colaba, Mumbai, India; b Department of Biotechnology, Abasaheb Garware College, Pune, India; c Indian Institute of Science Education and Research, Pune, India; d Department of Zoology, University of Pune, Ganeshkhind, Pune, India

**Keywords:** Microvillus inclusion disease, Intestine, Disease model, Zebrafish, Gut morphogenesis

## Abstract

Microvillus inclusion disease (MVID) is a life-threatening enteropathy characterised by malabsorption and incapacitating fluid loss due to chronic diarrhoea. Histological analysis has revealed that enterocytes in MVID patients exhibit reduction of microvilli, presence of microvillus inclusion bodies and intestinal villus atrophy, whereas genetic linkage analysis has identified mutations in *myosin Vb* gene as the main cause of MVID. In order to understand the cellular basis of MVID and the associated formation of inclusion bodies, an animal model that develops ex utero and is tractable genetically as well as by microscopy would be highly useful. Here we report that the intestine of the zebrafish *goosepimples (gsp)/myosin Vb (myoVb)* mutant shows severe reduction in intestinal folds - structures similar to mammalian villi. The loss of folds is further correlated with changes in the shape of enterocytes. In striking similarity with MVID patients, zebrafish *gsp/myoVb* mutant larvae exhibit microvillus atrophy, microvillus inclusions and accumulation of secretory material in enterocytes. We propose that the zebrafish *gsp/myoVb* mutant is a valuable model to study the pathophysiology of MVID. Furthermore, owing to the advantages of zebrafish in screening libraries of small molecules, the *gsp* mutant will be an ideal tool to identify compounds having therapeutic value against MVID.

## Highlights

• *myosin Vb* is expressed in the zebrafish intestine.
• *goosepimples/myosin Vb* function is essential for epithelial morphogenesis in the zebrafish intestine.
• The *goosepimples* mutant recapitulates pathognomonic features of microvillus inclusion disease.
• The function of *myosin Vb* in the intestine is conserved between fish and mammals.

## 1 Introduction

Microvillus inclusion disease (MVID) is a dreadful enteropathy inherited in an autosomal recessive manner and reported in infants often born of consanguineous parents. The main symptom of MVID is intractable diarrhoea and survival of the patients depends primarily on long-term parenteral nutrition. However, long-term parenteral nutrition may result in secondary complications such as bacterial infections, cholestatic liver disease and vascular as well as renal complications. The only successful MVID remedy so far has been bowel transplant (Bunn et al., 2000, Oliva et al., 1994, Ruemmele et al., 2004). This necessitates investigation of the cellular and physiological basis of the disease and possible remedies.

Recently, multiple studies have highlighted that a major cause of MVID are mutations in the *myosin Vb* gene (Muller et al., 2008, Ruemmele et al., 2010). An international patient registry reports 41 unique *myosin Vb* mutations thought to be associated with MVID (van der Velde et al., 2013). Myosin Vb is an actin based molecular motor. It is known to interact with Rab GTPases Rab11, Rab8 and Rab10 and functions in the transport of recycling endosomes and plasma membrane biogenesis (Lapierre et al., 2001, Liu et al., 2013, Roland et al., 2007). Consistently, it has been shown that in the enterocytes of MVID patients, loss of *myosin Vb* function results in the loss of Rab11-FIP5 positive recycling endosomes (Szperl et al., 2011). Furthermore, the apical transporter protein CD36 and basolateral transporters such as the Na^+^/K^+^ ATPase and transferrin receptor are mislocalised in MVID patients, suggesting that some aspects of enterocyte polarisation are compromised (Muller et al., 2008, Thoeni et al., 2014).

Studies involving the physiological and cellular characterisation of this disease depend on clinical explants obtained through biopsies of patients. The classic features of MVID at the tissue and cellular level include villus atrophy, reduction or shortening of microvilli and the presence of inclusion bodies in patient enterocytes (Golachowska et al., 2010). In addition to inclusion bodies, altered distribution of apical markers such as sucrose isomaltase (Ameen and Salas, 2000) and accumulation of Periodic acid-Schiff staining (PAS) positive material, presumably secretory granules (Phillips et al., 2004) have also been reported in the enterocytes of MVID patients. So far, the knockdown of *myosin Vb* using si-RNA in the Caco2 cell line has been used as a model for MVID (Ruemmele et al., 2010, Thoeni et al., 2014). Very recently, Myo5b knockout mouse models exhibiting attributes of MVID have been published (Carton-Garcia et al., 2015, Schneeberger et al., 2015, Weis et al., 2016). Other animal models for this disease include *Rab8, Rab11a* and *Cdc42* knockout mice, which also exhibit microvillus inclusions in the enterocytes (Sakamori et al., 2012, Sato et al., 2007, Sobajima et al., 2014). Nevertheless, a vertebrate model that is tractable by light and fluorescence microscopy would be an asset in understanding the cell biology of MVID and unravelling the role of Myosin Vb mediated trafficking in the intestinal epithelium.

The zebrafish intestine provides an excellent model to investigate intestinal development and disease. It is easily visible under a stereomicroscope through the transparent skin of developing larvae and shows gross morphological similarities with the human intestine. The similarities at the cellular, molecular and functional level are quite remarkable (Wallace et al., 2005). The intestine of a zebrafish exhibits folds, which are similar to mammalian villi, while its enterocytes exhibit apical microvilli just like mammalian enterocytes. It has been successfully established as a model system for intestine associated disorders such as inflammatory bowel disease (Oehlers et al., 2012, Oehlers et al., 2011). Thus, the zebrafish could provide a potential model to understand the development and progression of MVID and help find its remedy.

Here we report that the intestines of zebrafish *gsp/myoVb* mutant larvae exhibit the classic features of MVID. The mutant enterocytes have shorter microvilli and contain microvillus inclusion bodies very similar to MVID patients. Additionally, the shape of the mutant enterocytes is altered, which possibly affects the formation of intestinal folds. Furthermore, we demonstrate that the mutant larvae exhibit defects in lipid absorption. Our analyses presented here indicate that the function of Myosin Vb is conserved between fish and humans and that the zebrafish *gsp* mutant is an excellent animal model to study the cellular basis of MVID.

## 2 Results

### 2.1 *gsp/myoVb* is expressed in the developing zebrafish gut

**Fig. 1:**
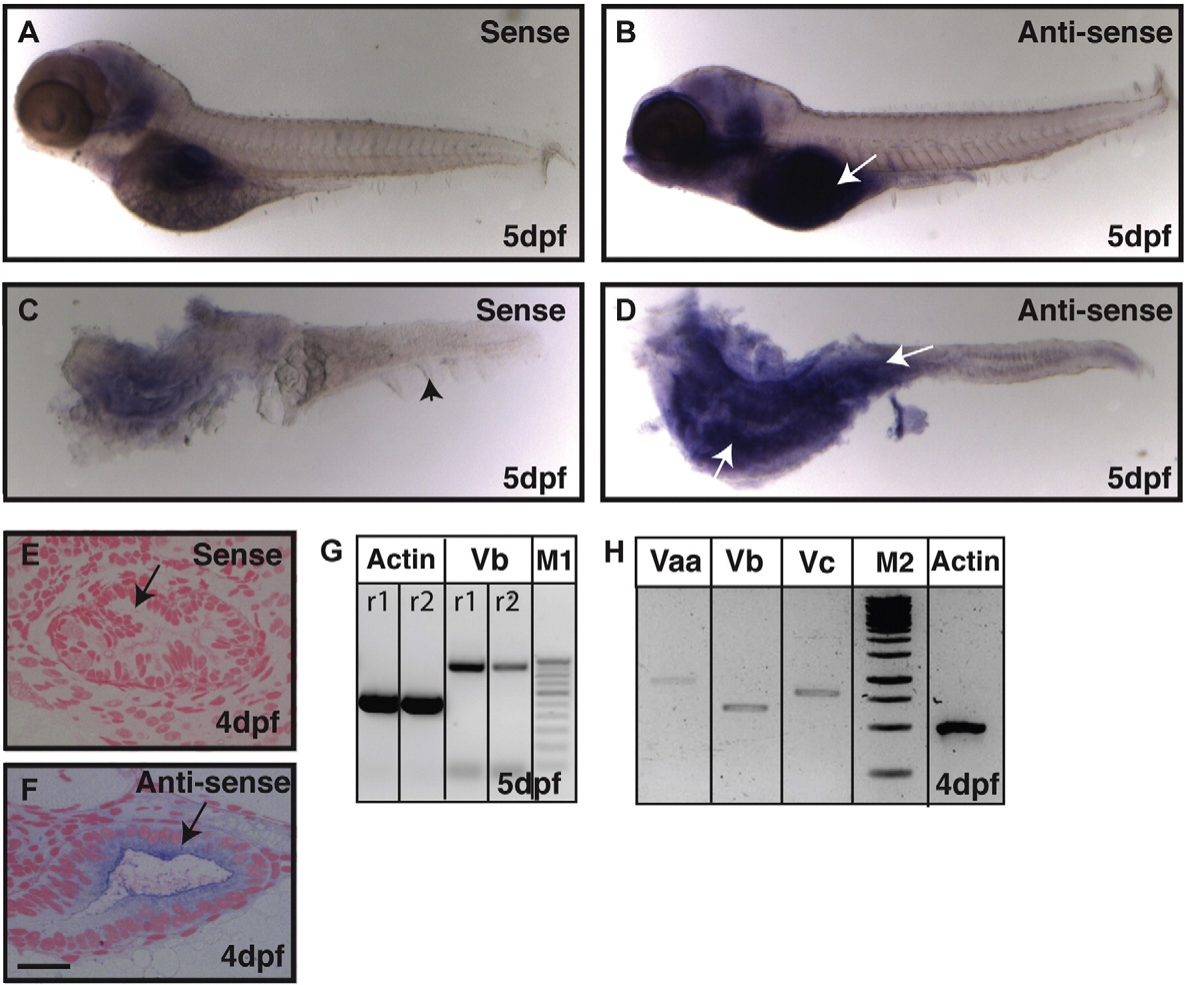
Expression analysis reveals that *gsp/myoVb* is expressed in the intestine of zebrafish larvae. In situ hybridisation analyses on 5-day-old larvae using sense control probe (A, C) and anti-sense probe against *myosin Vb* (B, D) in whole mount (A, B) and post-staining isolated intestines (C, D) reveal that *myosin Vb* is expressed in the intestine. The histological sections of 4dpf (E, F) larvae stained using sense control probe (E) and anti-sense probe (F) show the presence of *myosin Vb* transcripts in the gut epithelium. RT-PCR analyses performed on cDNA from intestines isolated from 4 and 5 day-old larvae show that *myosin Vb* (G) and other two *myosin V* paralogues, *Vaa* and *Vc* (H) are expressed in the intestine. Abbreviation: M1 - 100 bp ladder marker. M2 - 1Kb ladder marker. The white arrows (in B and D) point towards the expression of *myoVb.* The black arrowhead in C points to the median fin-fold. The black arrows in (E, F) indicate the gut epithelium. r1 and r2 are PCR reactions 1 and 2, respectively. Note that the difference between the *myo Vb* amplicon size in G and H is due to two different primer pairs used. Scale bar in F is equal to 20 μm in E, F.

In humans, mutations in the *myosin Vb* gene have been linked to MVID (Muller et al., 2008, Ruemmele et al., 2010). We asked if the function of Myosin Vb is conserved in the zebrafish intestine. To begin with, we checked the expression of *myoVb* in the developing zebrafish gut by performing RNA in situ hybridisation. Our analysis revealed that *myoVb* is predominantly expressed in the anterior part of the intestine or intestinal bulb at 5 days post fertilisation (dpf) (Fig. 1A–D). While considerable *myoVb* expression was also seen in the proximal part of the midgut, we did not observe its expression in the distal midgut and posterior gut using RNA in situ hybridisation (Fig. 1A–D). Sectioning of 4dpf stained larvae and RT-PCR analysis of intestines isolated from 5-day-old larvae, further confirmed the expression of *myoVb* in the gut epithelium (Fig. 1E–G). Another round of RT-PCR analysis revealed expression of two other *myosin V* paralogues in the zebrafish intestine along with *myoVb* (Fig. 1H).

We conclude that three *myosin V* paralogues are expressed in the developing zebrafish intestine. Furthermore, the expression of *myoVb* is observed in the intestinal bulb as well as in the proximal part of the midgut.

### 2.2 The loss of *gsp/myoVb* function leads to a severe reduction in intestinal folds and concomitant changes in the shape of enterocytes

**Fig. 2:**
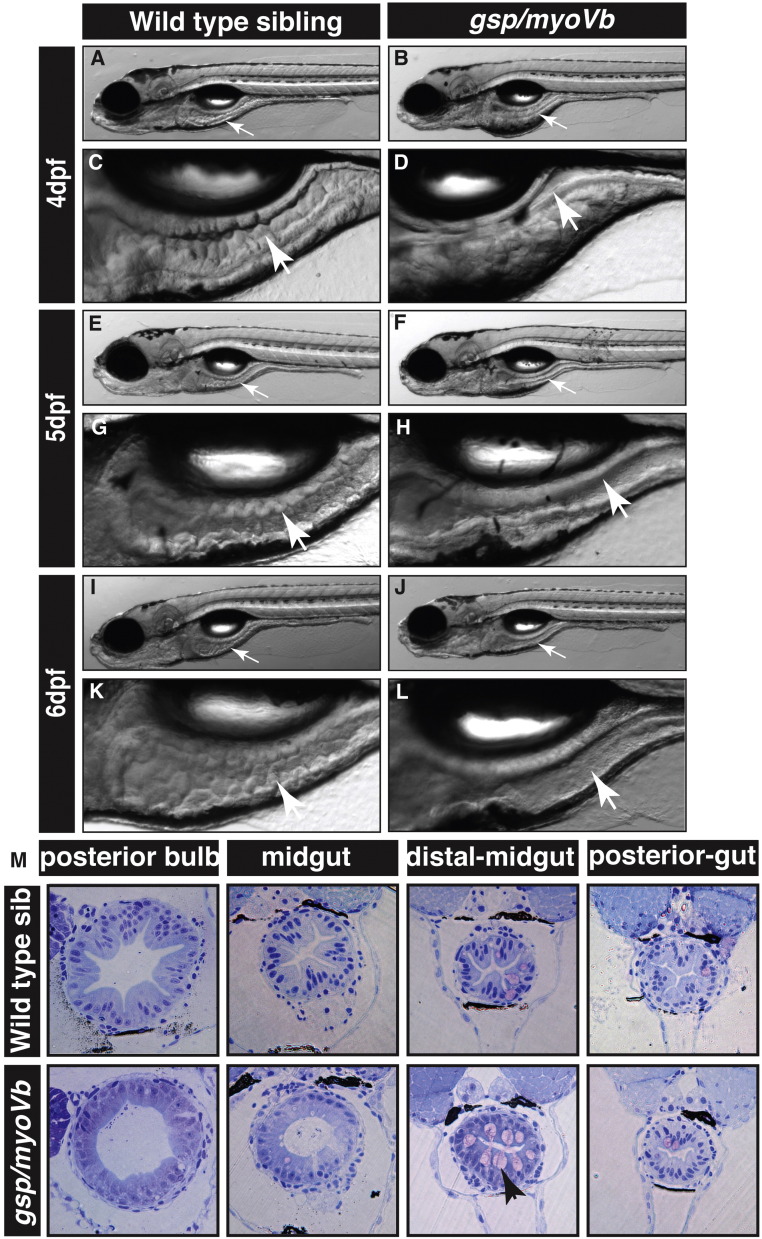
The zebrafish *gsp/myoVb* mutant exhibits a smooth intestine phenotype during 4 to 6 days post-fertilisation. The bright field images of developing zebrafish wild type larvae (A, E, I) and their intestines (C, G, K) exhibit rough intestinal walls indicating the presence of tissue folds. The *gsp/myoVb* mutant larvae (B, F, J) and their intestines (D, H, L) show smooth intestinal walls suggesting absence of intestinal folds. Arrows point to the intestine in all images. Histological sections (M) of different parts of the gut of wild type sibling and *gsp/myoVb* mutant. In total 4 wild type siblings and 4 mutants were analysed by histology. Abbreviation: sib = sibling.

Since *myoVb* is expressed in the intestinal epithelium, we asked whether there is a morphological phenotype in the intestine upon loss of Myosin Vb function. We have recently shown that the *gsp* locus, which is essential for the maintenance of epidermal architecture, encodes for the actin based molecular motor Myosin Vb (Sonal et al., 2014). While the morphological phenotype in the epidermis subsides after 3dpf, the mutants typically die at the beginning of metamorphosis due to unknown reasons. Therefore, we asked if the reason for death could be linked to an intestinal defect and investigated *gsp/myoVb* mutants. Careful observation under a stereomicroscope revealed altered morphology of the intestine in the *gsp/myoVb* mutants as compared to wild type larvae. As reported earlier (Ng et al., 2005, Wallace et al., 2005), in wild type larvae, intestinal fold morphogenesis started at 4dpf and subsequently the folds became more prominent (Fig. 2A,C,E,G,I,K). However, the mutant intestine lacked intestinal folds and appeared smooth-walled, even at 6dpf (Fig. 2B,D,F,H,J,L). These observations were further substantiated by histological analysis performed on 6-day-old larvae (Fig. 2M). The histological sections also revealed the presence of goblet cells in the distal part of the midgut, indicating proper gut differentiation in the mutant larvae.

**Fig. 3:**
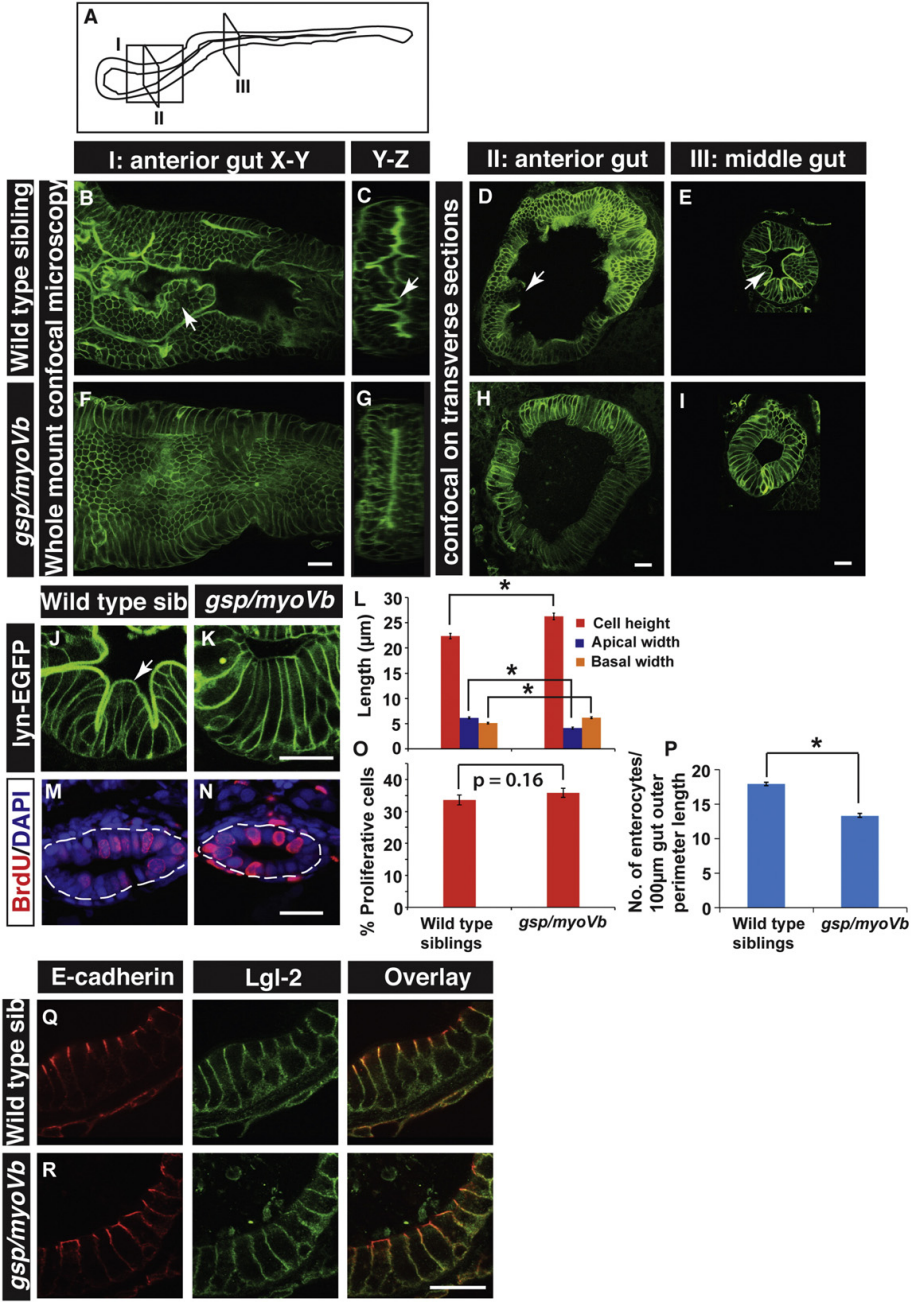
The loss of intestinal folds in *gsp/myoVb* mutants is accompanied by changes in cell shapes. A schematic showing various regions of the zebrafish larval gut (A). The boxed regions indicate the parts of the intestine where whole mounts were imaged (I) or intestines were sectioned (II, III). Confocal images of the whole mount (B, F), orthogonal sections (C, G), immuno-histological sections (D, E, H, I, J, K) of wild type sibling (B, C, D, E, J) and *gsp/myoVb* mutant (F, G, H, I, K) intestines stained for lyn-EGFP at 6dpf. Note the absence of intestinal folds in the *gsp/myoVb* mutants. The whole mount analysis was done on 4 siblings and 3 mutants whereas the immunohistology analysis was done on 9 siblings and 7 mutants. Bar graph (L) showing measurements of the height, apical width and basal width of enterocytes in wild type and *gsp/myoVb* mutant larvae at 6dpf. The enterocytes are taller and have a narrower apical domain in the *gsp/myoVb* mutants as compared to wild type. BrdU staining in wild type (M) and *gsp/myoVb* mutant larvae (N) at 3dpf followed by estimation of proliferation indices, which are represented in a bar graph (O). Number of enterocytes per 100 μm gut perimeter length was estimated from sections for 6-day-old wild type and *gsp/myoVb* mutant larvae and shown in a bar graph (P). Immuno-histological sections of zebrafish intestine in the wild type (Q) and *gsp/myoVb* mutant larvae (R) stained using anti E-cadherin and anti-Lgl2 antibodies. Arrows in B–E and J point to the intestinal folds. The square brackets show the comparison and asterisks indicate the statistically significant difference at p < 0.05 by Student's *t*-test. The error bars in L, O, and P represent SEM. Scale bars are equivalent to 20 μm. Abbreviation: sib = sibling.

To further confirm the effect on the formation of intestinal folds and to understand the possible cause of the phenotype, we asked whether the cell shape, cell proliferation and cell polarisation is perturbed in the *gsp* mutant enterocytes. To analyse the shapes of enterocytes, we sectioned the gut from 6-day-old wild type and *gsp/myoVb* mutant larvae in the background of Tg(*cldnB*:*lynEGFP*) transgenic line. In this transgenic line, the *claudinB* promoter drives expression of lyn-tagged EGFP in several epithelial tissues including the gut (Haas and Gilmour, 2006). We performed immunohistology on the wild type and mutant gut using an anti-GFP antibody. Corroborating the histology analysis, we observed a clear reduction in fold formation in the intestine of the *gsp* mutant larvae as compared to wild type (Fig. 3B–I). Furthermore, in wild type larvae, the crests of folds are formed by wedge-shaped enterocytes with expanded/broad apical surfaces facing the lumen and narrow basolateral sides facing the extracellular matrix. However, in the *gsp/myoVb* mutants, the enterocytes appeared more columnar as well as taller as compared to the wild type (Fig. 3J,K). We further measured the central height, apical width and basal width of intestinal cells from the histological sections of anterior, mid and posterior gut. This quantification confirmed that while wild type enterocytes (7 larvae, n = 88) are broader apically and narrower on the basal side, *gsp/myoVb* mutant enterocytes (9 larvae, n = 93) are apically narrower and basally broader (Fig. 3L; p < 0.001). Additionally, mutant enterocytes are taller than the enterocytes in wild type larvae (Fig. 3L; p < 0.001).

In addition to cell shape changes, cell proliferation could also help in shaping the intestinal folds. To test whether a reduction in cell proliferation contributes to the decrease in fold formation, we estimated the BrdU index in the wild type and mutant gut at 3dpf when intestinal epithelium is reported to be highly proliferative in the wild type (Ng et al., 2005, Wallace et al., 2005). Our analysis revealed no significant difference in cell proliferation in the mutant gut as compared to wild type siblings at 3dpf (Fig. 3M–O; p = 0.155). We further determined the cell density in the gut at a later stage by estimating the number of enterocytes per 100 μm of the gut perimeter at 6dpf. We performed this analysis mostly on the sections used to make the morphometric measurements mentioned above. This analysis suggests that the cell density decreases in the mutant gut by 6dpf (Fig. 3P; p < 0.005).

**Supplementary Fig. 1:**
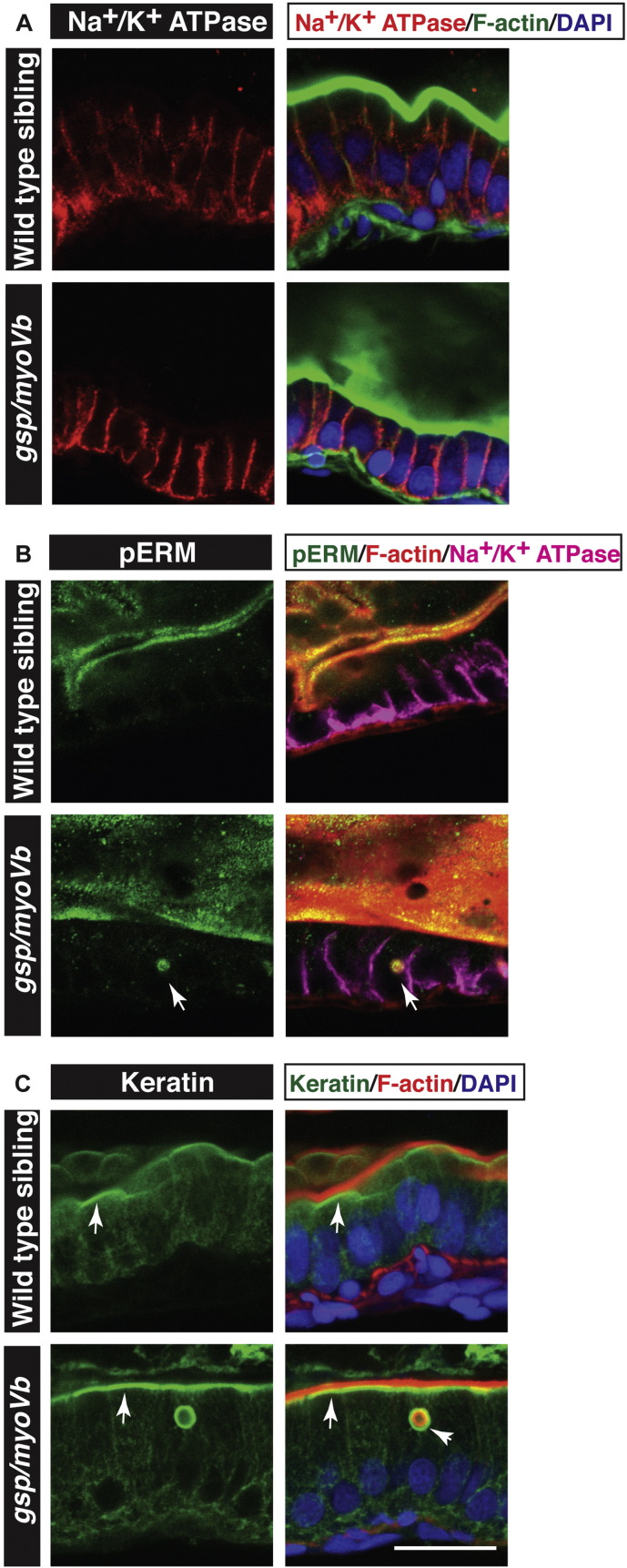
Characterisation of intestinal phenotypes using apical and basolateral markers. Immunohistology on 6 day-old wild type and *gsp/myoVb* gut followed by confocal microscopy to analyse localisation of Na^+^/K^+^ ATPase (A) pERM (B) and Keratin (C) along with other markers mentioned. Na^+^/K^+^ ATPase localises to the basolateral domain in wild type as well as *gsp/myoVb* mutant enterocytes (A). pERM (arrows in B) and Keratin (arrowhead in C) localise to inclusion bodies. Note the subapical localisation of keratin (arrows in C) in wild type as well as in *gsp/myoVb* gut. Scale bar = 20 μm.

Previous studies on MVID patient biopsies and the recent analysis of the *Myo5b* knockout mouse have indicated that certain transporter proteins, such as CD36, Na^+^/K^+^ ATPase and transferrin receptor, are mislocalised in the absence of Myosin Vb function, indicative of a partial disruption of apico-basal polarity in mature enterocytes (Ameen and Salas, 2000, Carton-Garcia et al., 2015, Muller et al., 2008, Thoeni et al., 2014). We asked whether cell polarity is disrupted along with cell shapes in the enterocytes of *gsp* mutant larvae. Since the loss of or perturbations in cell polarity affect adherens junction formation, we analysed the localisation of E-cadherin — a transmembrane core component of the adherens junctions — in enterocytes. We used Lethal giant larvae 2 (Lgl2) as a marker for the basolateral domain. In siblings, E-cadherin localises laterally and is enriched on the apical side of the lateral surface, whereas Lgl2 localises to the basolateral domain. We did not observe any obvious defect in the localisation of E-cadherin or Lgl2 in the *gsp/myoVb* mutant intestine (Fig. 3Q,R). In addition, immunohistological analysis using anti-Na^+^/K^+^ ATPase antibody did not reveal mislocalisation of Na^+^/K^+^ ATPase to the apical domain (Supplementary Fig. 1A).

To conclude, we show that in the absence of *gsp/myoVb* function, intestinal folds do not form properly. We propose that the effect of the loss of *gsp/myoVb* function on fold formation is manifested through a combination of change in the enterocyte morphology and reduction in enterocyte density. We further conclude that in the absence of MyoVb function, there are no gross defects in the polarity of enterocytes.

### 2.3 Enterocytes in *gsp/myoVb* mutant exhibit microvillus inclusions and other cellular features of MVID

**Fig. 4:**
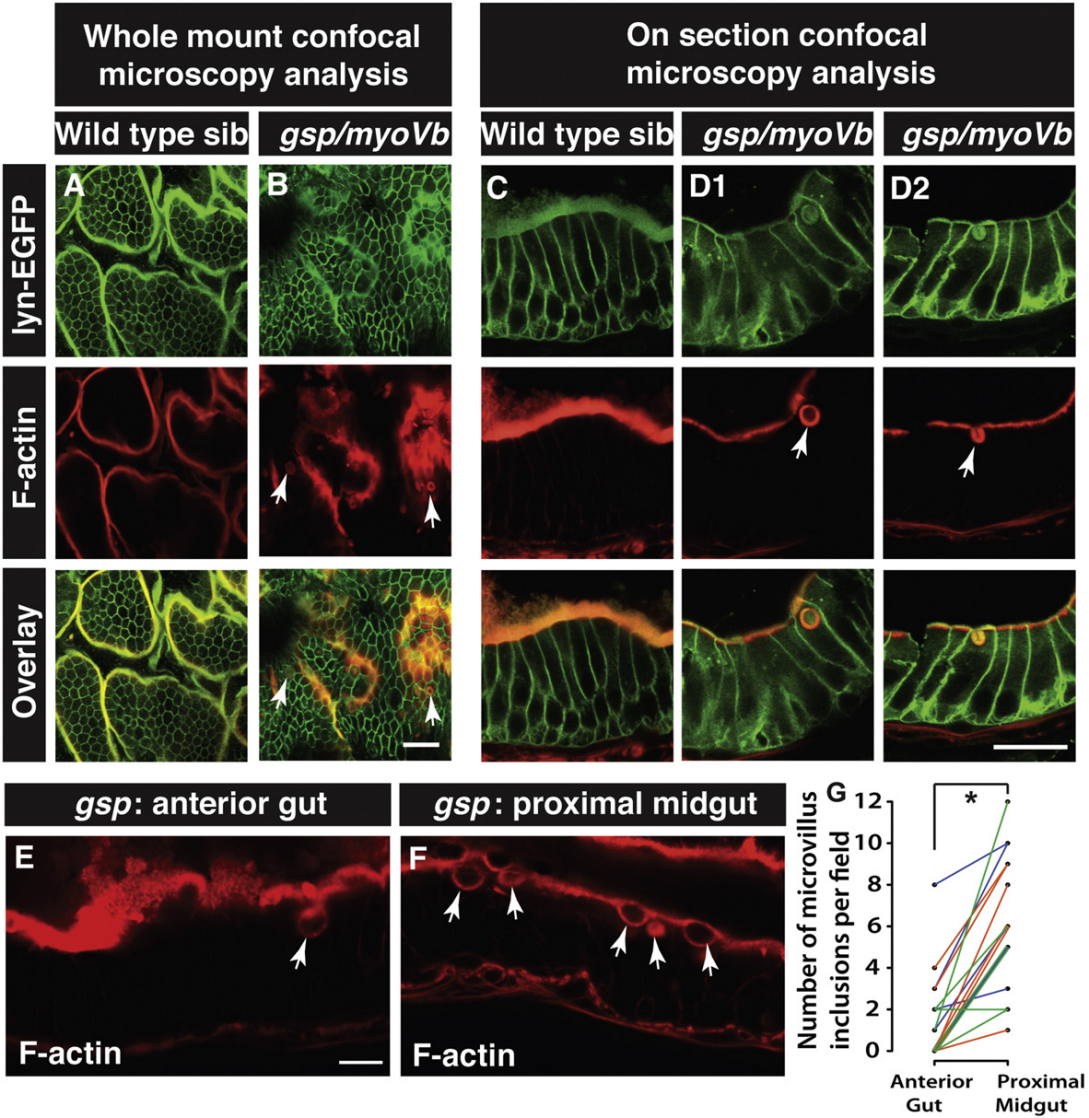
Enterocytes in *gsp/myoVb* mutants show presence of microvillus inclusions. Confocal .microscopy analysis of whole mount (A, B) and cryosections (C, D1, D2) of the anterior gut of wild type sibling (A, C) and *gsp/myoVb* mutant (B, D1, D2) larvae stained for lyn-EGFP and F-actin (phalloidin) at 6dpf. In all, 3 mutants and 4 siblings were examined in whole mounts whereas 7 mutants and 7 siblings were analysed by cryosectioning. Confocal sections of anterior gut (E) and proximal midgut (F) from the same *gsp/myoVb* mutant stained for phalloidin. In total 17 mutant animals from three different sets were analysed this way. The graph (G) shows the comparison between the number of inclusions in the anterior versus proximal midgut in mutants. Each line indicates one animal and different colours indicate values from different experimental sets. The arrows indicate phalloidin or lyn-EGFP labelled inclusion bodies in enterocytes. The asterisk represents statistically significant difference (p < 0.0001) by paired *t*-test. Scale bars: in B and D2 = 20 μm and in E = 10 μm. Abbreviation: sib = sibling.

Our results so far demonstrated a clear intestinal phenotype in the *gsp/myoVb* mutant. Therefore, we asked if the mutant enterocytes show the cellular attributes of MVID. Analysis of intestinal cells of MVID patients has shown the presence of large vesicular structures called inclusion bodies with microvillar components trapped within (Ameen and Salas, 2000, Reinshagen et al., 2002, Thoeni et al., 2014, van der Velde et al., 2013). Enterocytes in Rab8a, Myo5b knockout mice and Myo5b deficient Caco2 cells exhibit the presence of actin rings marked by phalloidin, a classic feature of MVID (Carton-Garcia et al., 2015, Ruemmele et al., 2010, Sato et al., 2007, Schneeberger et al., 2015, Weis et al., 2016). To check if such inclusion bodies are present in the enterocytes of *gsp/myoVb* mutant larvae, intestines of 6dpf larvae were stained with phalloidin. Both sibling and mutant intestines showed bright apical actin staining corresponding to microvilli or apical brush border of the enterocytes (Fig. 4A,B). Strikingly, a few *gsp/myoVb* enterocytes showed the presence of large phalloidin labelled subapical vesicular bodies (Fig. 4B). These inclusion bodies have a diameter of about 3–5 μm. Similar inclusion bodies were also seen when intestinal sections were stained with phalloidin (Fig. 4C–D2). The inclusion bodies consisted of F-actin and membrane. They were mostly associated with the apical membrane or were contiguous with the apical domain, suggesting their origin through invagination of the apical membrane (Fig. 4D2). Indeed, the microvillar component phospho-ERM (Ezrin-Radixin-Moesin), which connects the plasma membrane with the actin cytoskeleton (Algrain et al., 1993, Bretscher et al., 2002, Gautreau et al., 2000) was observed in the inclusion bodies along with F-actin (Supplementary Fig. 1B). Localisation of cytokeratins at the apical domain of endodermal cells has been reported during the onset of lumen formation in the developing zebrafish intestine at 2dpf (Ng et al., 2005). We asked whether any of the keratins are components of the apical submembrane cytoskeleton or terminal web (Ameen and Salas, 2000) in zebrafish enterocytes and whether this cytoskeleton is a part of the inclusion bodies. Immunohistology analysis using a pan anti-cytokeratin antibody (AE1/AE3) revealed that keratin cytoskeleton is present sub-apically, just below the actin cytoskeleton, in wild type as well as in *gsp* mutant enterocytes and that inclusion bodies are surrounded by the keratin cytoskeleton in the mutant enterocytes (Supplementary Fig. 1C).

We checked if there was a preponderance of these phalloidin rings in any particular region/s of the intestine. During initial inspection of phalloidin stained samples, rings were rarely observed in the posterior gut and the distal midgut (the region having a high density of goblet cells) so we restricted our analysis to the anterior gut and proximal midgut. Paired analysis revealed that phalloidin rings are more prevalent in the proximal midgut than in the anterior gut (Fig. 4E–G).

**Fig. 5:**
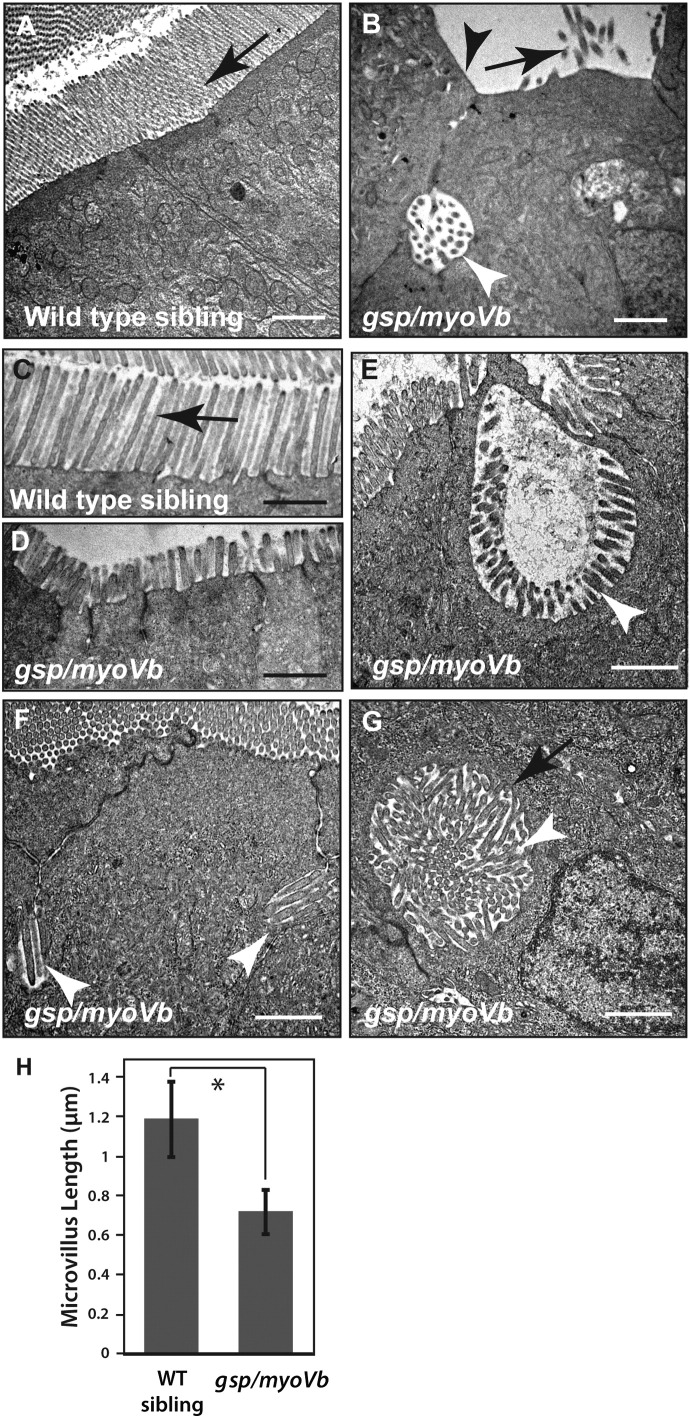
Shorter microvilli and microvillus inclusions, the main attributes of MVID, are present in enterocytes of *gsp/myoVb* mutants. Electron micrographs of thin sections, passing through the enterocytes of 6dpf wild type (A, C) and *gsp/myoVb* mutant larvae (B, D–G). Note the absence of microvilli (B), the reduction in microvillus length (D) and presence of microvillus inclusions (B, E–G) in *gsp/myoVb* enterocytes as compared to wild type enterocytes (A). The image shown in G is a high magnification image of the inclusion seen in Fig. 7I. The bar graph (H) shows the difference in the microvillar length in the midgut of wild type siblings and *gsp* mutants. For the EM analysis, we used 4 siblings and 5 mutant animals. The length measurements were done on 137 microvilli from 49 enterocytes of 4 siblings and on 95 microvilli from 33 enterocytes of 3 mutants. The white arrowheads indicate microvillus inclusions whereas the black arrows (in A-C) point to microvilli. A black arrowhead in (B) points to the loss of microvilli whereas a black arrow in (G) points to terminal web surrounding the microvillus inclusion. The square bracket shows the comparison and asterisk indicates that the difference is statistically significant by an unpaired *t*-test with unequal variance at p < 0.0001. The error bars in H represent the SD. Scale bars correspond to 1 μm.

To understand the ultrastructure of these inclusion bodies and the status of microvilli, we performed TEM analysis on the intestines of wild type and mutant larvae at 6dpf. In the mutant, we observed inclusion bodies containing microvilli trapped within (Fig. 5B,E,G), a pathognomonic feature of MVID. Microvillus inclusions in mutant enterocytes were observed at various subcellular locations such as at the apical side, at the lateral membrane domains and near the nucleus (Fig. 5B, E–G). Some of the inclusions were surrounded by a terminal web (Fig. 5G). Furthermore, when we compared the length of the microvilli in control and *gsp/myoVb* mutants, we observed that microvilli were significantly shorter in *gsp/myoVb* mutant enterocytes (Fig. 5A–D, H). We also noticed that the extent of the reduction varied in different mutant animals (Fig. 5A–D, H).

**Fig. 6:**
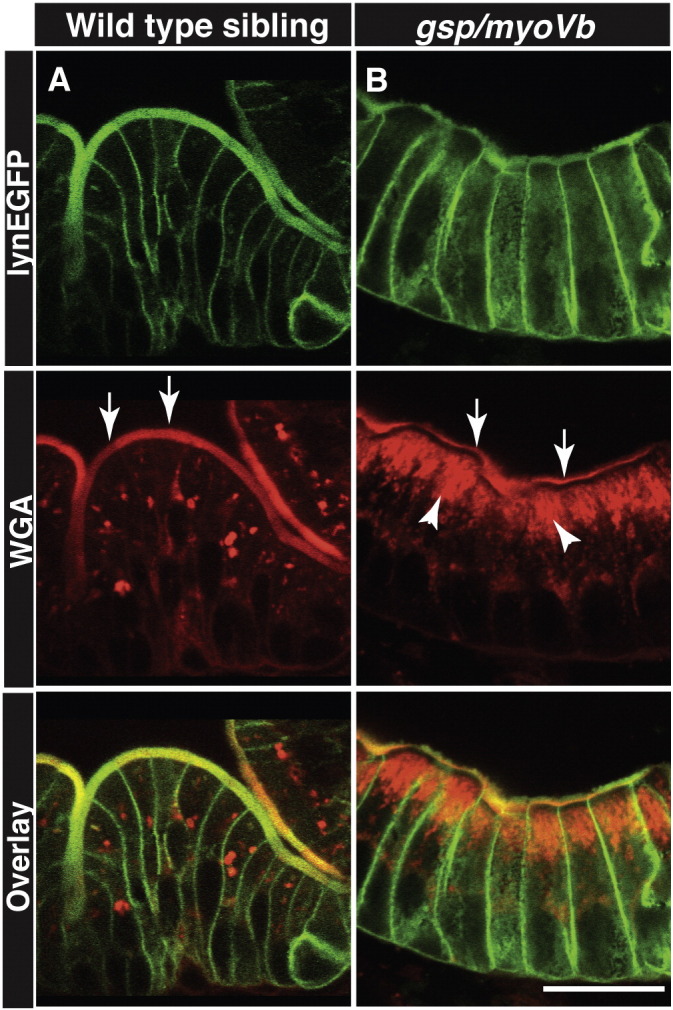
Secretory material accumulates in the enterocytes of *gsp/myoVb* mutant larvae. Immunohistology on 6dpf wild type siblings (A) and *gsp/myoVb* mutant (B) intestine using anti-GFP antibody and Alexa fluor 594 conjugated WGA. The white arrows point to the WGA labelling of apical domain of the wild type and mutant enterocytes whereas the arrowheads point to the intracellular staining. Note the considerable decrease in apical staining and concomitant increase in intracellular staining in *gsp/myoVb* mutant. For this analysis, we checked 3 siblings and 4 mutants by histology. The scale bar is equivalent to 20 μm.

Another prominent feature of MVID is the accumulation of PAS positive material, presumably secretory granules, in the enterocytes (Phillips et al., 2004). PAS or Periodic acid-Schiff staining typically detects carbohydrates and glycoproteins. We asked whether *gsp/myoVb* mutants exhibit any such accumulation of secretory granules in the enterocytes. Instead of using PAS, we used fluorescently labelled Wheat Germ Agglutinin (WGA), which also binds to carbohydrates and glycoproteins. Our analysis performed on sections revealed large accumulation of glycoproteins in the apical domain of enterocytes confirming MVID-like cellular phenotypes in the *gsp/myoVb* mutant larvae (Fig. 6A,B).

To conclude, histological as well as electron microscopy analyses show that *gsp/myoVb* mutant enterocytes exhibit important attributes of MVID, such as the presence of microvillus inclusions, reduction in microvillus length, and accumulation of secretory granules.

### 2.4 Absorption of lipids is diminished in the *gsp/myoVb* mutant intestine

**Fig. 7:**
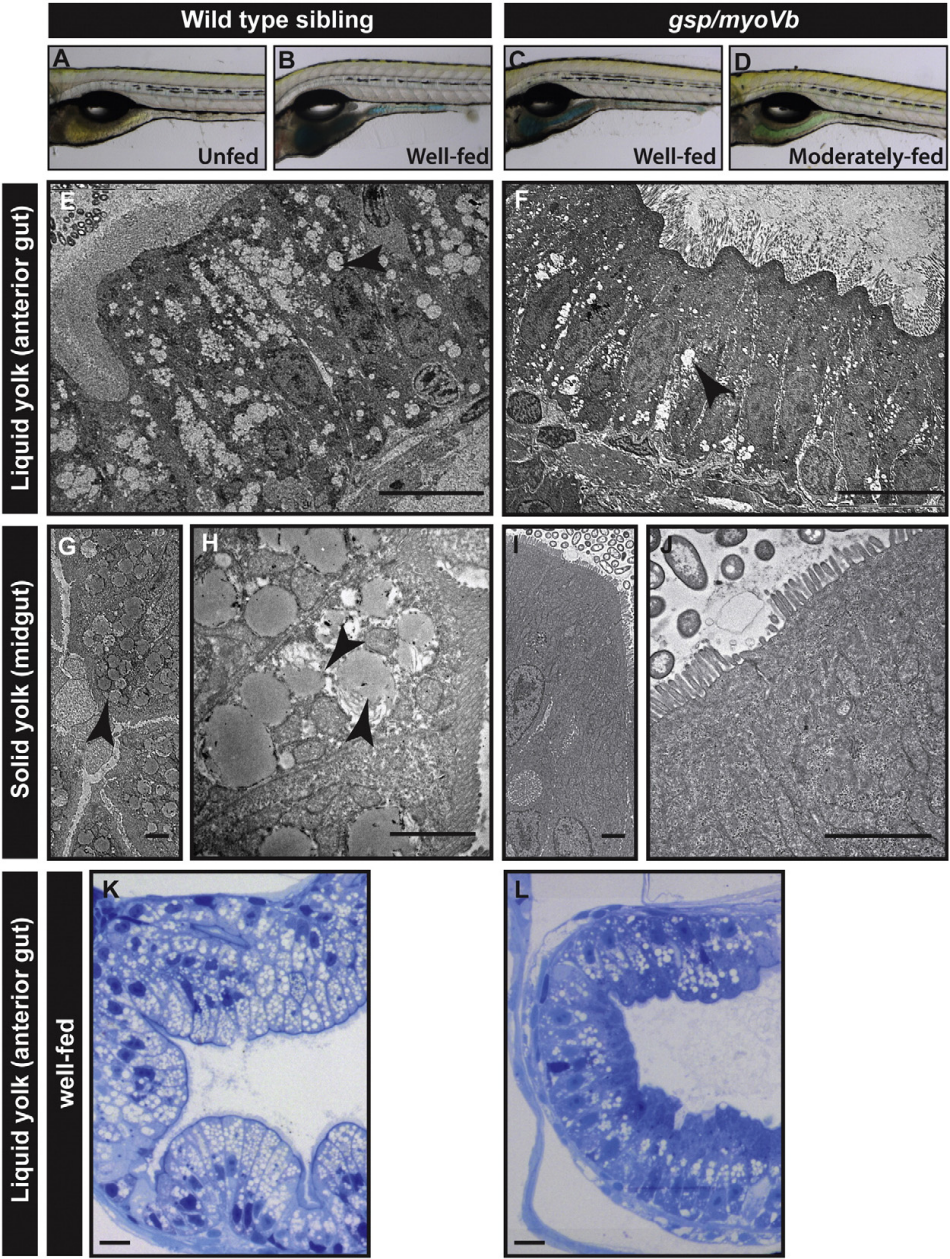
Lipid absorption is decreased in *gsp/myoVb* mutant larvae. Bright field images (A–D) of wild type (A, B) and mutant larvae (C, D), either unfed (A) or fed with coloured emulsified yolk (B–D). Electron micrograph of enterocytes from wild type siblings (E, G, H) and *gsp/myoVb* mutant larvae (F, I, J) fed with liquid yolk (E, F) and solid yolk (low magnification G, I; higher magnification H, J). Histological sections of the anterior gut of well-fed wild-type sibling (K) and *gsp/myoVb* (L) larvae. The micrographs of liquid yolk fed larval enterocytes were taken in the anterior part of the gut whereas the solid yolk fed larval enterocytes were imaged in the midgut. Note that while the absorption is low in the anterior gut in *gsp/myoVb* mutant larvae, it is almost absent in the midgut. The EM analysis was done on 3 wild type siblings and 3 mutants out of 9 animals of each genotype, which were additionally analysed by histology (not shown). In the second set, 3 well-fed siblings and 4 well-fed mutants were preselected and used for histological analysis. The representative images for this set are presented in K and L. The black arrowheads point towards the lipid droplets in the enterocytes. Scale bars: in E, F, K and L corresponds to 10 μm, in G, H, I, and J correspond to 2 μm.

Next, we asked if the cellular phenotypes in the *gsp/myoVb* mutant intestine also affect the absorptive function of the intestine. To test this directly, we incubated 6-day-old mutant and wild type larvae with either solid (boiled) or liquid (raw) egg-yolk, emulsified in fish water. To assess the uptake of food, an edible colouring agent was added in the yolk emulsion. We observed varied uptake of egg-yolk by both wild type siblings as well as by the mutant larvae (Fig. 7A–D; data not shown). In the next set of experiments, which did not involve the colouring agent, we assayed the fed and unfed larvae for the presence of lipid droplets in the enterocytes (Walters et al., 2012). Electron microscopy and histological analysis on wild type siblings fed with either solid or liquid yolk revealed striking accumulation of lipid droplets in the enterocytes. In contrast, the *gsp/myoVb* mutant enterocytes exhibited a mild to drastic reduction in lipid accumulation (Fig. 7E–J; data not shown). Despite the variations observed in the extent of feeding, there was a consistent reduction in lipid accumulation in the enterocytes of 9 mutant larvae, observed by histology (Data not shown). In the second set, we sorted the animals depending upon the consumption of coloured liquid yolk. Based on the colour intensity in the intestine, larvae were classified as ‘well-fed’ and ‘moderately-fed’ (Fig. 7A–D). Of these, three well-fed wild type siblings and four well-fed mutants were further examined by histology. This analysis clearly revealed that well-fed mutant enterocytes exhibited reduced amount of lipid droplets as compared to the well-fed siblings (Fig. 7K,L).

Our analysis convincingly proves that the loss of Myosin Vb function leads to a considerable reduction in lipid absorption.

## 3 Discussion

The association of *myosin Vb* mutations with MVID has been clearly established over the last few years (Muller et al., 2008, Ruemmele et al., 2010). For quite some time, there was no animal model to study the pathophysiology of MVID or to understand the mechanistic basis of the cellular phenotype shown by enterocytes in MVID patients. Until recently, depletion of Myosin Vb activity in Caco2 cells has been used as a strategy to recapitulate the cellular features of MVID (Ruemmele et al., 2010). Consequently, the exact function of *myosin Vb* in enterocytes has remained poorly understood. Here we report the zebrafish *gsp/myosin Vb* mutant as the first non-mammalian model for MVID.

Our analyses have revealed that the zebrafish *gsp/myoVb* mutant recapitulates morphological and cellular features of MVID. The gut morphology of *gsp/myoVb* mutant larvae exhibits drastic reduction in intestinal folds. This is strikingly similar to the villus atrophy reported in human MVID patients. Furthermore, we show that in the absence of Myosin Vb function, enterocytes exhibit microvillus shortening and microvillus inclusions, pathognomonic features of MVID. Our data also show that the occurrence of microvillus inclusions is higher in the midgut than in the anterior gut. Besides, they are rarely present in the posterior gut and the distal midgut. Some of the inclusions appear to be connected with the apical domain, suggesting their apical origin. In addition to inclusion bodies, we observed the accumulation of secretory material in the apical half of the enterocytes in *gsp/myoVb* mutant larvae. These features are similar to those shown by human MVID patients. Furthermore, regional differences in the occurrence of inclusions are also reported in a mouse knock-out model (Weis et al., 2016). Thus, the zebrafish model will very well complement the recently published mouse knockout models to unravel the mechanistic basis of acquisition of disease status by *myosin Vb* deficient enterocytes (Carton-Garcia et al., 2015, Schneeberger et al., 2015, Weis et al., 2016) Our analyses further indicate the conservation of the function of *myosin Vb* between fish and mammalian enterocytes. Although two other paralogues, *myosin Vaa* and *myosin Vc,* are expressed in the intestine, they do not seem to compensate for the loss of *myosin Vb* function in the proximal or anterior part of the intestine. The absence of microvillus inclusions in the distal part of the intestine might be a consequence of functional differences within the intestinal epithelium and/or due to redundant functioning of the myosin V paralogues in the distal part of the intestine.

In the mouse Myo5b knockout model, the reduction of blood glucose levels has been linked with watery diarrhoea and defects in absorption (Carton-Garcia et al., 2015). We used the egg-yolk feeding assay (Walters et al., 2012) to directly investigate the effect of *gsp/myoVb* mutation on lipid absorption. While both wild type and mutant larvae ingested yolk, some of the mutant larvae showed reduced uptake of yolk as compared to the wild type. We observed considerable reduction in lipid droplets in the enterocytes of well-fed mutants. This reduction in lipid accumulation indicates decrease in absorption of nutrients by the mutant enterocytes.

One of the novel findings of this study is the effect of loss of *myoVb* on the shape of enterocytes. Our analysis shows that wedge-shaped cells - apically broader and basally narrower - are associated with the intestinal folds in zebrafish larvae. In the absence of Myosin Vb function, the cells remain apically narrower and basally broader. In addition, their density decreases by 6dpf. Our data suggest that the function of Myosin Vb is important to achieve the cell shape change and maintenance of enterocyte number. Differential growth due to restricted cell proliferation has been proposed to be an important factor in the process of vilification (Shyer et al., 2013). We propose that the absence of fold formation is a consequence of improper enterocyte shape and inadequate cell density in the *gsp/myosin Vb* mutant. At this stage, it is not clear whether the decrease in enterocyte density in the absence of Myosin Vb function is due to reduction in enterocytes number. Alternatively, the decreased density could be a consequence of the differential packaging of cells due to altered cell morphology. Further investigation is essential to address this issue.

The effect on cell shapes warranted analysis of enterocyte polarity. However, we did not observe any gross effect on the apico-basal polarity of the enterocytes by analysing localisation of apico-lateral and basolateral markers of polarity such as E-cadherin, Lgl or Na^+^/K^+^ ATPase. The earlier studies on human biopsies, mouse knockout models and CaCo 2 cell lines are quite inconsistent with respect to localisation of E-cadherin and Na^+^/K^+^ ATPase (Ameen and Salas, 2000, Carton-Garcia et al., 2015, Schneeberger et al., 2015, Thoeni et al., 2014, Weis et al., 2016). Our results are in line with the reports that suggest no effect on E-cadherin and Na^+^/K^+^ ATPase (Ameen and Salas, 2000, Carton-Garcia et al., 2015). The effect on the basolateral markers could be a secondary phenomenon and becomes apparent at a later disease state or developmental stages giving rise to such inconsistencies in localisation studies. However, it is clear that the effect on cell polarity is manifested as a mislocalisation or reduction in localisation of apical components like CD36, transferrin receptor (TfR), CD10, alkaline phosphatase (ALP), Ezrin, pERM (Muller et al., 2008, Thoeni et al., 2014). In line with this, we also observed localisation of pERM to the phalloidin ring. The microvillus inclusions are also surrounded by keratin or the terminal web, suggesting the presence of an aberrant apical domain within the cell. Further investigation is needed to check the status of other apical markers in enterocytes of zebrafish *gsp/myoVb* mutant larvae.

MVID is a fatal genetic disease. The therapies available include parenteral nutrition and bowel transplants (Bunn et al., 2000, Oliva et al., 1994, Ruemmele et al., 2004). Long-term parenteral nutrition may lead to complications such as bacterial infections, cholestatic liver disease and renal complications. In the absence of a suitable animal model, the progress on identifying small molecules that might help to alleviate the disease condition has not been possible so far. Over the last 20 years, the zebrafish has emerged as a powerful model to study human diseases and disorders (Ingham, 2009). Zebrafish disease models are useful to understand the pathophysiology of a disease and to screen libraries of small molecules that may have therapeutic value (Peterson and Fishman, 2011). The zebrafish *gsp/myosin Vb* mutant reported here would be an invaluable tool for screening such libraries to identify compounds that will help manage the disease better and improve the lives of MVID patients.

## 4 Experimental procedures

### 4.1 Fish strains

Three alleles of *goosepimples (gsp)* viz. *gsp*^*NS042*^, *gsp*^*K38B*^ and *gsp*^*AT21*^ were used for the morphological observations of the gut. However, all the analyses presented in this paper were done using *gsp*^*NS042*^ allele. The transgenic line for *cldb:lyn-egfp* (Haas and Gilmour, 2006) was crossed to *gsp*^*NS042*^ to obtain EGFP labelling of the intestinal epithelium in *gsp/myoVb* mutants. For in situ hybridisation, embryos from the *albino* strain were used. For zebrafish maintenance and experimentation, the guidelines recommended by the Committee for the Purpose of Control and Supervision of Experiments on Animals (CPCSEA), Govt. of India, were followed.

### 4.2 RT-PCR and in situ hybridisation

To check gene expression, intestines dissected out from 4 and 5 dpf larvae were used. RNA was extracted using a trizol (Invitogen, USA) based method. cDNA synthesis was carried out using up to 1 μg RNA using a cAMV reverse transcription kit (Invitrogen, USA). The following primers were used for PCR: *myosin-Vaa* (Forward: 5′gaacaaggagaaccgttcca3′; Reverse: 5′gtacgcaggagaaccaggag3′), *myosin-Vb* (Forward1: 5′ acgagagacaacgatatcag 3′; Reverse1: 5′cttgttgagttgacgatttgg 3′; Forward2: 5′acgagaatctggctgttgct3′; Reverse2: 5′gcagcaggttgtagccatct3′), *myosin-Vc* (Forward: 5′ acgggctcaagggttagaaa3′; Reverse: 5′gcttgaagctgctcgttctc3′) and *β-actin* (Forward: 5′aaggccaacagggaaaagat3′; Reverse: 5′aagtggtctcgtggataccg3′).

Using primers, a region of *myosin Vb* (NM_001161632.1; bp 2967–3866) - with low homology to the other two paralogues - was amplified and cloned into pCR TOPOII (Invitrogen, USA). Templates were linearised to synthesise probes using either T7 or SP6 RNA polymerase from DIG RNA Labelling Kit (Roche, Switzerland). In situ hybridisations were performed using a protocol described earlier with a few modifications (Nüsslein-Volhard and Dahm, 2002).

### 4.3 Immunohistology

For whole intestine staining, larvae were first fixed overnight at 4°C in 4% paraformaldehyde (PFA). After PBS washes the digestive tracts of the larvae were dissected out. The intestinal tissue was permeabilised with 0.8% PBT (0.8% Triton X-100 in phosphate buffer), blocked with 10% Normal Goat Serum (NGS) (Jackson ImmunoResearch; USA) in PBT for 2 h.

For staining intestinal sections GFP positive larvae were fixed in 4% PFA and kept overnight at 4 °C. For E-cadherin staining larvae were fixed in Bouin's fixative. The fixed larvae were upgraded to 30% sucrose and embedded in 14 cryomatrix blocks. 16 μm thin sections were cut using a cryotome (Leica, Germany). The sections were placed on poly-l-lysine coated slides. The slides were air-dried and the matrix was removed by distilled water and PBS washes. These sections were blocked with 10% NGS for 3 h.

The whole intestinal tissue and cryosections were incubated overnight in anti-E-cadherin antibody (1:100; BD Transduction Labs, USA; #610182), anti-GFP antibody (1:200; Torrey Pines Biolabs, USA; #TP401), anti-Lgl2 antibody (1:400; (Sonawane et al., 2009), anti-Na^+^ K^+^ ATPase (1:100; DSHB; #a5, deposited by Fambrough, D.M.), anti-pEzrin (T567)/Radixin (T564)/Moesin (T558) (1:50; Cell signalling technology; #3141S), anti-pan Cytokeratin AE1/AE3 (1:100; Abcam; ab27988), anti-BrdU (1:50; Acris antibodies, #SM1667PS), Alexa Fluor 594 conjugated WGA (5 μg/ml; Invitrogen, USA; #W11262), Rhodamine Phalloidin (1:40; Invitrogen, USA, #R415) or Atto 647N-Phalloidin (1:400; Sigma, USA; #65906) in 1% NGS in PBT. After PBT washes, wherever appropriate, Alexa 488 (1:250; Invitrogen, USA), Cy3 (1:750; Jackson ImmunoResearch, USA) and Cy5 (1:750; Jackson ImmunoResearch, USA) conjugated secondary antibodies against mouse and rabbit IgG were used in 1% NGS in PBT for 4 h. The tissue or sections were washed in PBT, post-fixed in 4% PFA for 30 min and mounted in glycerol or vectashield (Vector Labs, USA) for imaging.

### 4.4 Egg yolk feeding assay

This assay was performed as previously described (Walters et al., 2012) with a few modifications. 20 larvae each of the mutants and siblings were placed in 2 ml E3 buffer in separate wells in a 6 well plate. Either 600 μl raw or 600 mg of boiled egg yolk was mixed well with E3 to obtain a homogenous emulsion of 2 ml egg yolk stock. Whenever essential 100 μl food colour (GFC, batch no. 040/14, India), containing 1% brilliant blue FCF, propylene glycol, potable water and permitted diluent, was added to the stock. One milliliter of this stock was added to each of the two wells containing larvae to get 10% egg yolk solution in E3. The larvae were incubated with yolk for 3 h, split into 2 groups of 10 larvae each, one of which was fixed for electron microscopy and the other for histology. The control group was left unfed and treated in the same way.

### 4.5 Histology and electron microscopy

For histology, 6dpf larvae were fixed in 4% PFA at room temperature for 30 min and then overnight at 4 °C. They were dehydrated in ethanol and embedded in Epon-Araldite. 1 μm sections were cut with a wet glass knife on a Leica Ultracut UC6 Ultramicrotome. Sections were lifted onto 1% gelatin coated slides and stained with Richardson's Stain (Richardson et al., 1960) at 60 °C for 2 min followed by thorough washing with water. Slides were mounted with DPX and imaged on a Zeiss Axioskop 2 plus, (Zeiss, Germany) with a Nikon Digital Sight DS-Fi2 (Nikon, Japan). The animals stained for whole mount in situ hybridisation were processed the same way as described above but sections were cut at 4 μm or 10 μm thickness, stained with 1% Eosine yellowish (Merck 1345) in tap water for 30 min and briefly rinsed with distilled water, mounted in distilled water and imaged using AxioCam MRc5 on Zeiss Apotome microscope.

For electron microscopy, 6dpf larvae were fixed in PFA and glutaraldehyde 2.5% each in 0.1 M sodium cacodylate buffer (Electron Microscopy Sciences, Cat#15949) at room temperature for a half hour followed by overnight incubation at 4 °C. They were washed 3 times in 0.1 M phosphate buffer (PB) at pH 6.2, post-fixed in cold 1% osmium tetroxide (Sigma, 60H0150, USA) in 0.1 M PB at pH 6.2 for 45 min, washed thrice in distilled water, en bloc stained in 0.5% aqueous uranyl acetate (Electron Microscopy Sciences, Cat#22400, USA) for 1 h, again washed 3 times with distilled water, dehydrated in ethanol and embedded in Epon-Araldite. 70–100 nm sections were cut with a diamond knife (DiATOME, USA) on a Leica UC6 ultramicrotome (Leica, Germany) collected on slot grids, post-stained with lead citrate and imaged in a Zeiss Libra 120 TEM (Zeiss, Germany).

### 4.6 BrdU labelling and immunostaining

At 70.5hpf, larvae were incubated with 10 mM BrdU in 5% DMSO in E3 medium for 30 min at 28.5 °C followed by 3 washes in E3 and incubation in E3 for 1 h. All the above steps were done with E3 without methylene blue. The larvae were then fixed in 4% PFA for 30 min at room temperature and overnight at 4 °C followed by incubation in 30% sucrose in PBS overnight and embedding of 5–7 larvae using O.C.T. Compound (Sakura Tissue Tek, 4583). Sections of 40 μm thickness were cut using a Leica CM1510-1 cryostat, collected serially in individual wells of a 12 well plate in PBS After placing first 12 sections in 12 wells, the 13th section was collected in the 1st well and the process was continued.

All sections from the well containing the maximum number of sections and the 6th well from it, were collected together in a 2 ml microcentrifuge tube. The sections were treated with 2 N HCl for 30 min at room temperature, washed 3 times with PBS followed by 6 washes for 10 min in PBT and blocking with 10% NGS in PBT for 4 h at RT. The sections were processed for immunohistology by incubating them successively with anti BrdU antibody and secondary antibody with DAPI (10 μg/ml) for 24 h at 4 °C on a rotor with 3 washes in between with PBT. After incubation with secondary antibody and DAPI the sections were washed 3 times in PBT, fixed in 4% PFA for 30 min, cleared in a glycerol series to 70% glycerol in PBS.

### 4.7 Image acquisition and processing

For imaging immunostainings of whole intestines, the samples were mounted in 80% glycerol by placing a coverslip on them. Whole mounts and Vectashield (Vector Laboratories, USA) mounted cryosections of intestine were imaged using the Zeiss LSM 510 Meta or Zeiss LSM 710 with EC Plan-Neofluar 40 ×/1.30 oil objective or Plan-Apochromat 63 ×/1.40 oil (Zeiss, Germany). 1024 × 1024 image dimensions were used, with an averaging of 4 or 16. For estimation of inclusion bodies, imaging was done on Zeiss 510 meta with imaging conditions: 63 ×/1.4 oil, 1.5 × zoom, 1024 × 1024 image dimension with scaling X,Y: 0.093 μm, scaling Z: 0.419 μm, 11–30 stacks.

For imaging of BrdU stainings, sections were mounted in glycerol and imaged on either a Zeiss LSM 510 or Zeiss LSM 5 exciter using either a Plan-Apochromat 63 ×/1.40 oil or LCI Plan-Neofluar 63 ×/1.30 water objective (Zeiss, Germany). Optical sections were taken every 1.5 μm. Every 4th optical section was counted for DAPI and BrdU using the cell counter plugin on FIJI and repeated counting of the same nucleus in different slices was avoided. 3 independent sets of experiments were performed and quantitated.

For imaging phenotypes, the embryos were treated with MESAB and mounted on Methyl Cellulose gel. Imaging of the morphological phenotype and in situ hybridisation reaction was done on Zeiss SteREO Discovery with AxioCam (Zeiss, Germany).

Either ImageJ (Schneider et al., 2012), Fiji (Schindelin et al., 2012) or ZEN Light Edition 2009 was used for image processing.

### 4.8 Morphometric measurements of enterocytes, estimation of microvillus inclusions, cell proliferation index and data analysis

Confocal scans of cryosections of anterior, mid and posterior gut stained for lyn-EGFP were used for measurements of the central height, apical width and basal width of enterocytes. For each enterocyte a confocal slice was selected where the cell appeared the broadest. To quantify the cellular dimensions, lines corresponding to the height, apical and basal width were traced and measured using Measure tool of Image J (Schneider et al., 2012). Around 90 cells from 7 to 9 larvae were analysed for wild type sibling and *gsp/myoVb* mutants each.

For analysing the average number of cells per basal area, the basal perimeter of the intestinal epithelial layer was traced and measured for the confocal sections of the cryosections using Fiji (Schindelin et al., 2012). The cell number per unit perimeter was calculated as the ratio of number of cells facing the lumen side in a section to its basal perimeter. It was normalised to 100 μm perimeter. The analysis was done on 16 sections from 7 mutant larvae and 23 sections from wild type siblings.

The BrdU analysis was done on sections from a total of 15–17 larvae from 3 independent sets, counting a total of 56 sections in the siblings and 61 in mutants.

For estimation of the relative proportions of inclusion bodies, isolated intestines were phalloidin stained. Confocal stacks of whole mounts of the same frame size were taken, one each for the ventral wall of the anterior gut and the proximal midgut of the same animal. Actin rings showing a central clearing were counted from an equal number of slices of equal thickness from the two stacks of the same animal using the cell counter plugin on ImageJ (Schneider et al., 2012). Repetitive counting was avoided by keeping track of inclusions in previous stacks. The data was collected from 5 to 6 animals from three different experiments.

For wild type siblings, the lengths of a total of 137 microvilli were measured from 49 enterocytes from the midgut of 4 animals using ImageJ (Schneider et al., 2012). For mutants, we estimated length from 95 microvilli from 33 midgut enterocytes of 3 animals.

The data was plotted using MS-Excel and Adobe Illustrator and compared by a paired or unpaired Student's *t*-test where appropriate using MS-Excel.

The following is the supplementary data related to this article.

## Competing interests statement

The authors declare no competing financial interests.
